# Doing implementation research on health governance: a frontline researcher’s reflexive account of field-level challenges and their management

**DOI:** 10.1186/s12939-017-0695-7

**Published:** 2017-11-15

**Authors:** Gupteswar Patel, Surekha Garimella, Kerry Scott, Shinjini Mondal, Asha George, Kabir Sheikh

**Affiliations:** 1 The University of Newcastle, Callaghan, NSW 2308 Australia; 2 Public Health Foundation of India, Gurugram, Haryana 122002 India; 3 Johns Hopkins School of Public Health, Baltimore, 21205 USA; 4 McGill University, Montreal, H3A 0G4 Canada; 5 University of the Western Cape, Private Bag X17, Bellville, 7535 South Africa

**Keywords:** Implementation research, Frontline researcher, Reflexivity, Experiences, Challenges, Management, Resilience

## Abstract

**Background:**

Implementation Research (IR) in and around health systems comes with unique challenges for researchers including implementation, multi-layer governance, and ethical issues. Partnerships between researchers, implementers, policy makers and community members are central to IR and come with additional challenges. In this paper, we elaborate on the challenges faced by frontline field researchers, drawing from experience with an IR study on Village Health Sanitation and Nutrition Committees (VHSNCs).

**Methods:**

The IR on VHSNC took place in one state/province in India over an 18-month research period. The IR study had twin components; intervention and in-depth research. The intervention sought to strengthen the VHSNC functioning, and concurrently the research arm sought to understand the contextual factors, pathways and mechanism affecting VHSNC functions. Frontline researchers were employed for data collection and a research assistant was living in the study sites. The frontline research assistant experienced a range of challenges, while collecting data from the study sites, which were documented as field memos and analysed using inductive content analysis approach.

**Results:**

Due to the relational nature of IR, the challenges coalesced around two sets of relationships (a) between the community and frontline researchers and (b) between implementers and frontline researchers. In the community, the frontline researcher was viewed as the supervisor of the intervention and was perceived by the community to have power to bring about beneficial changes with public services and facilities. Implementers expected help from the frontline researcher in problem-solving in VHSNCs, and feedback on community mobilization to improve their approaches. A concerted effort was undertaken by the whole research team to clarify and dispel concerns among the community and implementers through careful and constant communication. The strategies employed were both managerial, relational and reflexive in nature.

**Conclusion:**

Frontline researchers through their experiences shape the research process and its outcome and they play a central role in the research. It demonstrates that frontline researcher resilience is very crucial when conducting health policy and systems research.

## Background

Implementation research (IR) is a growing field of inquiry within the broader terrain of health policy and systems research (HPSR) that seeks to better our understanding of how decisions about health policies, programmes, and practices are made [1]. It does so by examining the process of health policy and program implementation in real-world contexts. Implementation is the process of carrying a plan into outcome; in health research these plans take the form of policies or programs [1]. The IR canvas includes different aspects of implementation, such as contextual factors and the implementation processes themselves, the results of implementation, and how to promote a program’s large-scale use and sustainability. The intent of IR is to understand what, why, and how interventions work in real word settings and to test approaches to improve them [1]. IR contends with a range of challenges, including ethical issues [2], determining how best to account for contextual features when evaluating implementation outcomes [3], multi-layer problems of implementation, which are attributed to the fact that several layers of government are often involved in policy processes [4, 5] and drawing policy-relevant conclusions from research using few cases with many variables [5, 6].

Partnerships between researchers, implementers, policy makers and community members are central to IR. For example, researchers can collaborate with community members to identify neglected local issues, uncovering shortfalls in health systems performance, and leading to increased accountability of healthcare organisations [1]. The collaborative and flexible nature of IR, and the frequent use of qualitative methods in this field demands extensive researcher reflexivity. Although, reflexivity should be an integral element of research sensibility and a key component of methodological rigour, it is more common in social science disciplines than public health [7, 8]. There are extensive philosophical interrogations and reflections on what reflexivity is and why it is important. At a very simple level it involves reflecting on the approach through which research is carried out and understanding how the process influences the outcome [9]. It also involves reflecting on the research process using approaches that demonstrate self-consciousness and consideration of the researcher’s presence, role, and impact. Hence, reflexivity involves components of acknowledgment and identification as well as critical evaluation [10].

In this paper, we share the experiences and challenges faced by frontline researchers (research assistant’s is the first author of this paper) at the field level, who were involved in an 18 month IR study of a governance intervention, specifically, the establishment of Village Health Sanitation and Nutrition Committees (VHSNCs) in India. This intervention component, which involved the implementation of VHSNC guidelines, and was implemented by a non-governmental organization (NGO), was accompanied by a parallel research component understanding the processes influencing the intervention’s implementation. The research component was conducted by an independent research team, including a frontline research assistant responsible for coordinating data collection at the field level. Reflexive practice was central to the functioning of the research team and allowed for constant feedback and improvisation at the field level. Memos written by the research assistant were a practical way to document and deal with challenges on a daily basis, allowing reflection at various levels of the team.

In HPSR processes generally, including IR, one of the least understood spaces is the interaction between frontline research assistants and the people they interact with at the data collection sites, and how that can influence research process and the data that is collected. It is important to understand those interactions because the field level researchers are the key mediators of any research study for the people at the field level [11]. Despite the important role that frontline workers play [12] there is not much available in the form of studies or reflections about these workers and the challenges that they face, especially in the field of IR and HPSR [11, 12]. It is much more common to find reflections and intellectual debates around this issue in social science disciplines, such as anthropology. For example, Gupta (2014), writes that the role of a research assistant is fundamental to configure the process and result of data collection [13].

In this paper, we focus on the frontline research assistant’s experiences related to the challenges faced during data collection for the research arm of this study, and discuss our management strategies to overcome these challenges in relation to frontline researchers’ roles, perceived locus of power, and expectations from co-research team members and other stakeholders. We also highlight the crucial importance of appropriate, flexible and responsive management strategies and practices in facilitating IR projects. While we recognise that everything cannot be planned in advance, it is important that in the planning stages of IR, possible field level challenges are taken into account and strategies put in place to guide responses to them.

## The implementation research study

This IR study took place in a rural area of northern India, within 250 kilometres of Delhi and had twin components happening concurrently: intervention and in-depth research. The intervention sought to strengthen the functioning of VHSNCs through contracting a local NGO to implement a government-designed VHSNC support package. The support package involved community mobilization to raise awareness of the VHSNC and to invite people to join, forming or expanding VHSNCs to include at least 1 members from a range of social groups (caste and religious) and women and men from these groups as well from the local health system. The mobilisation was followed by the training of the new VHSNC members, and then supporting VHSNC meetings and activities. The support package was implemented in 50 VHSNCs in 50 villages. The village residents were primarily farmers and migrant labourers, along with few households of government servants. Villages were connected through roadways and could be visited using mostly two-wheelers, since the roads were too poorly maintained for other vehicles. Government schools, Anganwadi centres (Peripheral facility for children’s pre-school education and nutrition), and health facilities were severely understaffed and poorly maintained; most had no running water, and some had no electricity. The research component, meanwhile, sought to understand the contextual factors, pathways and mechanisms facilitating or hindering the intervention’s implementation. It explored actor perspectives on VHSNC composition, processes, and functions, community embeddedness of the committees, and the activities taken up by VHSNCs in real world settings.

In-depth longitudinal qualitative research was conducted in four of the 50 intervention villages, which were selected purposively keeping in mind the characteristics of distance from the health centre and diversity of people living in the village in terms of caste and religion, and marginalisation. More specifically, the data were collected in three waves over the 18-month period. In total, 74 in-depth interviews (IDIs) and 18 focus group discussions (FGDs) were conducted with VHSNC members, community non-VHSNC members, NGO staff, health system actors (health services administrators, Primary Health Centre (PHC) supervisors, ASHAs and ANMs) and community health and nutrition workers (called Anganwadi workers). Data collection also involved observations of 54 intervention processes and VHSNC activities consisting of monthly meetings, quarterly cluster level meetings, community mobilisation, VHSNC member training and NGO staff training.

### Implementation team

The intervention implementation team comprised seven NGO staff: three facilitators (one for each cluster), one senior manager, two assistant managers, and one project director. The three cluster facilitators were in their early 30s. One was a man with a post-graduate degree in social work, and two were women who had married into local families and resided with their in-laws. Both of the female cluster facilitators had completed their secondary education. The senior manager and two assistant managers were in their mid-40s. They had worked for eight to 15 years in the education and development sectors. The project director was in her 40s, and she had post-graduated in library science, law, and public administration. All implementation team members were employed by the NGO, and the three cluster facilitators were employed only for the VHSNC strengthening program. The NGO had an office in the small town within the intervention site and their head office was in the state’s capital city. All the implementers except the project director were native to the local area; the project director was from another region of the state.

### Research team

The research team included members from several academic and non-governmental institutions; five members of this team (the principal investigator, co-investigator, research coordinator, research associate and research assistant) visited the field on a regular basis. The research assistant lived in a small town in the research area during the study period and collected the majority of the data. The research associate and research coordinator visited the study sites regularly and held debriefing with the research assistant weekly, in which they reflected on the data collection processes and discussed emerging themes, logistics, and next steps.

## Methods

Qualitative research is a social process of negotiations starting from conceptualisation to entering, staying and exiting the research site [14]. Field memos are widely recommended as a means of documenting contextual information as well as researchers’ ideas, insights and experiences [15]. Writing field memos are integral elements of qualitative research designs to facilitate preliminary coding, increase rigor and trustworthiness, provide context to inform data analysis and foster researcher reflexivity among other uses [16–19]. In general field memos remain of interest mainly to the research team and their content and insights are necessary for analysis of primary data and are rarely treated as data that can be analysed and shared in themselves [20–24].

### Dataset and analysis for this paper

Over the 18-month period of the IR study, the frontline research assistant experienced a range of challenges while collecting the data from the study sites. These experiences were documented as field memos. A total of 39 memos were written during this period and have been analysed for this paper. These memos documented the experience of conducting longitudinal qualitative implementation research, including questions from community members and the implementing NGO staff about the study and researchers.

An Inductive content analysis approach was used to analyse the field memos. Inductive content analysis is used when the aim is to describe a phenomenon and where there are no previous studies dealing with this phenomenon [25–27]. This approach was adopted since very little is known regarding the interactions between frontline research assistants and communities where IR takes place.

**Fig. 1:**
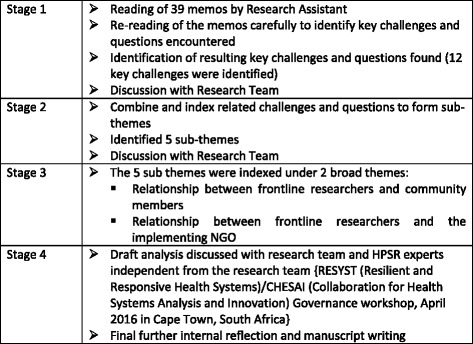
Stages of analysis of memos

The memos were read, re-read, interpreted and acted on for their meanings and the challenges and questions were derived directly from the memo texts. The stages of analysis are described in Fig. 1. The first stage of the analysis process involved the research assistant’s (the first author of this paper) reading and re-reading the memos. Following which core challenges faced in the field by the research assistant and questions posed by different actors were identified. These challenges and questions were discussed with the research team and further discussions with the second author of this paper were held which allowed for further reflection on these experiences and questions. The next stage involved combining and indexing of related challenges and questions to form sub-themes. The sub-themes are identified by bringing together components or fragments of the challenges and questions. Through this process five sub themes were identified and discussed with the research team. In the final stage the five sub themes were indexed under two broad overarching themes (1) Relationship between frontline researchers and community members and (2) Relationship between frontline researchers and the implementing non-government organization (NGO). A draft of the analysis presented in this paper was discussed with the team and with HPSR experts independent from the research team. A final further round of reflection among the research team took place and the manuscript was written. This process of combining internal, external and further internal reflections helped in coalescing the many sides and aspects of experiences. While no new specific insights were generated, the process allowed for a nuanced reflection of the experiences and strengthened the themes intrinsic in the embedded challenges when using an IR approach.

## Results

Themes that emerged out of the analysis of and reflection on the memos are arranged in two parts: first, challenges and opportunities that arose between the researchers and community members, and second, challenges and opportunities that arose between the researchers and NGO staff. Both of these themes point to the relational nature of the IR process and the influence that ‘context’ and ‘perception of the powerful other’ can have on the day-to-day functioning of the research project, and the impact it can have on understanding, contextualising and interpreting the data/information that is generated from a project such as this.

## Relationship between frontline researchers and community members

In this section, we describe the perceptions of the community about the identity and influence of the research assistant, and their concerns about the observational activities of the research assistant. There was a common perception that the research assistant was a senior staff member of the implementing NGO who supervised the implementation work done by NGO facilitators. In addition, some also saw the research assistant as an influential outsider who could bring beneficial changes in their villages.

### Researcher as NGO staff member

While in our protocol the intervention and research components were conceptualised separately and were independent of each other, in the real world it was very difficult for the community to understand this difference. This difficulty in separating the two components was mirrored in the community’s perception that both the implementation and research teams were the same.

When visiting the villages, and at the beginning of each IDI and FGD, the research assistant and the other researchers (when present) introduced themselves as affiliated with a Delhi-based organisation and not the implementing NGO. The respondent information sheet, which was read out before each interview and focus group, also attempted to explain the research team’s affiliation. The research assistant sought to explain that the implementation and research teams were separate and that the two groups had distinct purposes: the research was to learn how the new VHSNC guidelines affected VHSNC functionality, and to understand enablers and barriers to VHSNC functionality and health system responsiveness. But this confusion remained right through till the end of the research period, despite constant engagement and communication explaining the difference.

Many community members saw the researchers and the NGO staff as part of a single group of professionals visiting the villages and working on VHSNCs. The perception of similarities between the frontline researchers and NGO staff may have been grounded in their common higher educational status and continued use of written materials, their common interest in VHSNCs, and the fact that on many occasions, the frontline research assistant travelled to the villages with the NGO staff. At first, the researchers visited the villages with the NGO staff in order to be introduced to the community.

“Research Assistant: Where is <name of a VHSNC member’s> house? I want to meet him [for interview] today.”

VHSNC member: Achha… [Okay], go straight and from that hand pump take left…… and then opposite to that… [Provided the direction to reach the VHSNC member’s house]

Non-VHSNC community member: Why are you asking? Who is he [addressing VHSNC member]?

Research Assistant: I am…

“VHSNC member: *Arey…* He is a sir. Working on swasth samiti [VHSNC in this context], he is from DDL [name of the NGO]” (Memo 23, Date: 28-03-2015)

Although the NGO staff were local to the region and the researchers were not, neither the NGO staff nor the researchers actually lived in the intervention villages. Moreover, higher-level NGO staff from other parts of the state also occasionally visited the villages to oversee the implementation of the intervention.

Community members particularly wondered if the frontline research assistant was a higher-level NGO staff member who had come to supervise the NGO facilitators. Community members identified our observation of activities as checking and supervising whether or not the VHSNC training, community mobilization and VHSNC support activities were being conducted correctly. We also asked the community members about the intervention as part of our research, this was perceived by members of the villages as an effort to check whether or not the NGO staff adequately performed their role. An issue that complicated our effort to collect data was that some members of the community were reluctant to speak openly about implementation issues because of their perception that we were staff of the NGO.

Are you their [NGO implementers’] supervisor? Because you come with them [for meetings] and write many things in your notebook and you also ask us about their work. If they make any mistake, are you going to lodge a complaint against them or fire them from their jobs? (Memo 18, Date: 19-03-2015)

Whenever the research assistant became aware of respondents who thought he might be supervising the NGO staff, he tried to explain that he was not supervising the staff, and the data collection has nothing to do with reporting about their performance to senior NGO staff. In this way we tried to allay the concerns expressed by some of the community members.

### The influential outsider

As researchers, we considered the VHSNC intervention as a means of generating collective benefit to the community. The research assistant attempted to explain, verbally and through the participant information sheet, that participating in the IDIs and FGDs was voluntary and would not bring participants any direct benefit. However, the continuous expectation that the frontline researchers, and especially the research assistant, would help remained. Some community members appealed to us to bring about beneficial changes in the public services and facilities in their villages because they perceived us as outsiders with the power to influence systems. They also expressed hope that we would solve the problems that they shared with us in the IDIs or FGDs.

Are you going to provide some extra services in our village? (Memo 24, Date: 29-04-2015)

Are you going to solve problems of the village? If not, what is the value of discussion about all problems of our village? (Memo 13, Date: 20-05-2015)

The VHSNC intervention was attempting to strengthen VHSNC functioning so that local issues such as drinking water, timeliness in public health services, immunisation, presence of doctor and nurses in PHCs, and medicine availability in public health facilities could be addressed. These are issues that cannot be solved in a short time and need persistent engagement with stakeholders. The benefits were not immediately apparent to the community. They were looking forward to immediate gains expecting, for example, the immediate release of untied funds. (The untied fund was an amount of 10,000 INR for each VHSNC provided by the National Rural Health Mission, government of India. The VHSNC members have the autonomy to spend the fund for the common benefits of villagers. The untied fund was a high priority interest of almost all VHSNC members, because they felt that several local issues could be solved at the village level using that fund.) Another benefit that they were looking for was the appointment of doctors and nurses. They expected that the researchers as powerful outsiders could make this happen.

The research assistant consistently tried to engage with the community on the limits of his and his team’s influence on such matters and recorded the following in his notebook:

I told them “The NGO is working to strengthen the VHSNC in 50 villages here, including yours, and we are only looking at how the VHSNCs are getting strengthened (if they are). We discuss in detail about VHSNCs with people, NGO facilitators and health system workers as well. So it is important for us to learn from you people rather than documenting our opinions” (memo 13, 21 Feb 2015).

I explained “We will not make changes in the village level public facilities and services. We are here to understand the VHSNC from your perspective since you [community] are living in the village and looking closely at the VHSNC. If you want any help to change in the public services then you can speak with the NGO staff [implementer] and they would be able to assist you” (memo 21, 17 April 2015).

This difference in the reality of limited power of the research team and the heightened expectation from the community was a constant source of discomfort for the team. A lot of time has to be dedicated to keeping this dialogue open and constantly convincing people about the researchers’ limitations and role. The research assistant, due to his interactions on a daily basis, bore the brunt of unresolved expectations from the community. There were a few people who understood what the research team tried to convey and did not hinder the data collection process, but some tension remained throughout the data collection process.

### Non-participatory observation is socially unacceptable

An important component of the research study was the observation of VHSNC activities (VHSNC meetings, trainings, and community mobilisation) as non-participant observers. We were seeking to identify the successes of the intervention, and the challenges it faced, in order to inform the policy at scale. We were not supposed to provide any inputs, and did not see ourselves as competent in the areas of community mobilization and training, unlike the NGO, which had this expertise. We sought to minimize our influence on the implementation of the intervention.

At the ground level, members of the community felt uncomfortable with having the research assistant present during these interactive social events, but not actively participating in them. There was also discomfort with having someone observing the group and its activities and writing things down.

During training, you keep on watching us and writing something. What do you write about us, and why do you do that? (Memo 36, Date: 23-05-2015)

This discomfort and confusion about why the research assistant was just watching and writing (rather than speaking and helping alongside the NGO staff) led some community members to speculate that research assistant was there to supervise the NGO activities, as discussed above. In addition, some people suggested that the research assistant should participate in the implementation, because as educated outsiders we had the capacity to provide useful inputs.

In this training, you seem like very educated person but why are you not teaching or speaking anything to us? (Memo 16, Date: 22-02-2015)

Observing without participating sometimes led to people believing that we lacked concern for the community, and were withholding knowledge that may be useful for them in dealing with their problems.

When these issues came up, the research assistant tried to manage this by explaining the distinction between the researcher and trainer roles. He also tried to make people understand the purpose behind keeping notes from the observed events. The following excerpts from his field memos illustrate how he explained the purpose of non-participant observation:

I told him [a VHSNC member]: “We observe to understand the VHSNC support process. We do this because you will work in the VHSNC according to how you are trained. Your understanding about the committee will directly or indirectly affect the VHSNC’s functionality. So, it is important to understand what was taught to you and how that knowledge helps you in your village. So that the same thing [implementation strategies] would be applied across the country, [and] decision makers at the country level would be aware [of those process and factors]”. (Memo 36, Date: 23-05-2015)

The staff from the NGO is responsible for delivering the training to you, and in case you have queries related to VHSNC or you want to learn more, you can ask them directly. (Memo 16, Date: 22-02-2015)

While these explanations seemed satisfactory to some people, others remained unsure about the purpose of non-participant observation by the research assistant. The ones who were satisfied did not ask any further questions and allowed the observation to take place and the others too let him be possibly because they were used to seeing him on a regular basis. These interactions highlight the vulnerable position of frontline researchers (in this case the research assistant). Throughout the data collection process there remained a level of uncertainty in our minds whether or not members of the community actually understood the research assistant’s role. Management of these queries and expectations therefore necessitated responses from the team mainly on the go and could not be managed beforehand.

## Relationship between frontline researchers and the implementing NGO

In this section, we describe how the staff of the NGO reacted to the research assistant’s presence in the field. These included the perceptions about him and the expectations they had from him in his capacity as someone from a big organization in Delhi.

### An additional hand to help in the intervention

The NGO staff quickly developed a comfortable relationship with the research team and specifically the research assistant, and became accustomed to having him at all their VHSNC activities. They often sought his help as another pair of hands, or as someone who could provide advice and inputs. The research assistant was not an implementation expert and had been discouraged from providing advice or feedback to the NGO, so as not to influence their activities or cause them to see him as a judgemental outside viewer.

Activities such as VHSNC monthly meetings and community mobilisation were significant components of the intervention. The NGO was responsible for organising and supporting these components, including providing support for VHSNC action for addressing local problems. But, many a time the implementers faced difficulties in the implementation of the intervention and viewed the researchers as people who could suggest and guide them in this process. Over the period of implementation of the intervention these types of situations emerged numerous times, and posed a difficult challenge for us. This was partly due to our close working circumstances, and the personal, professional and organisational relationship with NGO staff. The research assistant mainly faced this challenge because he was living in the same small town as some of the NGO staff, and it was close to the implementation sites. The following two excerpts from the research assistant’s memos describe situations where the NGO staff turned to him as an expert, despite his having no relevant training or mandate to provide inputs:

“In this village, ASHA [female community health activist] is there [selected] but not working because her training has not been conducted yet. So, in the ASHA’s place, can the trained Dai (traditional birth attendant) do the work and get the incentive from the ASHA program? If yes, what is the procedure to do that?” (Memo 9, Date: 11-12-2014)

Since you are accompanying us in community mobilisation, give your feedback and tell us what improvement is required. (Memo 7, Date: 14-11-2014)

The research assistant managed not to intervene in the implementation while maintaining positive relations by emphasising that he was at an early stage of his career with no experience in relation to organising community mobilisation and conducting village level meetings.

“I am a young researcher who has never organized community mobilization, hence I’m not at a good stage to comment on the event, however I really enjoyed documenting it”. “The implementation and administration of health programs are different in different states, so I have no clue how to solve this issues in this state”. (Memo 7, Date: 14-11-2014)

The NGO staff accepted the responses supportively and went ahead in the meetings and other events.

### Research assistant as an NGO supervisor

Similar to community members’ perception that the research assistant was observing the NGO staff, the NGO staff also brought up this concern. The in-depth research study required constant observation of the activities being conducted under the intervention component and the research assistant was expected to document the process of implementation as a part of research. Funding for the intervention was also provided by the research organisation and a national agency. At the ground level this translated into a perception among the NGO staff that he had a supervisory role over them. The NGO staff also felt that since he represented a funding agency, his observations could impact the intervention implementation process as well as the reputation of their NGO. These concerns were enduring and manifested in the form of the NGO staff’s asking him to write about their activities and efforts in a good light. For example, when efforts to organise trainings or meetings failed and not enough people came to them, there were tentative discussions among them to request the research assistant to change details in his documentation. However, they finally did not follow through with these requests. The following quote exemplifies these types of conversations as noted in the research assistant’s memo:

We implementers have planned for training today but failed to bring people. What to do? Out of 33 only 6 are here as of now. We will have to answer to higher people for this incident. Here, he [the research assistant] is also present from funding agency and he has written 6 numbers of participant in his note. Now what will we do? Go, touch his [research assistant’s] feet and request him to make the number at least 20. (Memo 29, Date: 19-06-2015)

To manage and alleviate their concerns the research assistant sought to explain the situation as follows:

“Although I am associated with the organisation who provides some fund to you, I am not here to monitor your work. I am only here to do the research work which requires documentation of every process that you follow while working on VHSNCs. So, feel free, since my documentation will not affect your reputation.” (Memo 29, Date: 19-06-2015)

Repeated explanations about the purpose of observation and documentation were necessary to disabuse the NGO staff of the perception that they were being supervised and judged. This points to the difficulty in practice of separating research and intervention when they are part of the same study, and also the difficulty faced by researchers in explaining complex research designs.

## Discussion

Through this reflexive account of frontline researchers’ experiences in the field during an 18-month long IR study seeking to strengthen VHSNCs in rural area of North India, we have described the challenges that arise when doing this type of research and the management strategies that we adopted to overcome them. Complex research designs are difficult to conceptualise, plan and execute. In addition, execution of these research designs in the real world comes with challenges linked to clarity of roles, power associated with different identities and positions that the researchers are perceived to represent, and maintaining the integrity of the research process due to these realities. In addition, our account also highlights the crucial role that frontline researchers, especially research assistants responsible for data collection, practicing ethics, maintaining relationships and transparent research practices, addressing people questions and expectations, play in configuring the research process through their daily encounters and experiences in the real world and the data that are generated and its interpretations [13].

As explained earlier, the IR study had two components - an intervention and an in-depth research study - which sought to understand contextual factors, pathways and mechanisms facilitating and hindering the intervention. It explored actor perspectives on VHSNC composition, processes and functions; community embeddedness of the committees; and the activities taken up by VHSNCs in real world settings. The study was designed in a particular way so that the research did not feed back into the intervention as the research component of the IR study was intended to understand the implementation process and context influencing the intervention in real world settings. While, in design, the components had to be autonomous to maintain the integrity of the research, executing this separation in the real world was much more complicated. Throughout the research process, we were constantly faced with having to explain the autonomous nature of the research and intervention components to the people in the community as well as the staff of the NGO. Study respondents, members of the communities, and the staff of the NGO (responsible for the implementation of the intervention) all found it difficult to differentiate between the study’s twin components, and to understand the nuances of the need for this separation.

In our research journey we had to deal with community members’ perceptions of us as powerful outsiders who could bring about beneficial changes in the community. In addition, the staff of the NGO who were responsible for the implementation of the intervention also saw us as powerful people who could impact the implementation process as well their reputation among community members. Managing these expectations required honesty about our limited power to influence the implementation process and the systems functioning. While we were successful sometimes in convincing people about our limited roles and power, at other times we were left in an ambiguous space of not knowing what people were feeling and how this would impact our data collection. Therefore, interactions between different sets of actors in IR teams need to be considered in the context of local conditions and means available to these actors to articulate their opinions. While, managing ‘what to do’ and ‘what can be done’ will be mediated by specific circumstances, internal reflections and deliberations within the research team need to be a constant activity to make sense of and act on constraints and possibilities.

## Conclusion

IR is fundamentally a social process involving interactions between people, implementation, and contexts. Therefore, frontline researchers through their encounters and experiences shape the research process and its outcomes. Research assistants responsible for data collection, practising ethics, maintaining relationships and transparent research practices, addressing people’s questions and expectations play a very crucial and central role in this process. Dealing with the challenges associated with social processes requires resilience and imagination in frontline researchers so that the integrity of the research process and data generated through this process is maintained. This can be done through the adoption of reflexive principles to guide research practices. Being reflexive allowed us to understand and deal with the challenges by reflecting on the elements of confusion and tension that are part of the researcher’s role and identity. We were able to draw upon a range of strategies to overcome these challenges by acknowledging that some field level dilemmas may not be easily resolved. We believe that to do rigorous IR there is a need for research teams to be flexible and reflexive in their approach in order to deal with challenges that are bound to come with the complex research designs that are normally associated with this field. We demonstrate that frontline researcher resilience is very crucial when conducting health policy and systems research.

## Abbreviations

ANM: Auxiliary Nurse Midwife
ASHA: Accredited Social Health Activist
FGD: Focus Group Discussion
HPSR: Health Policy and Systems Research
IDI: In-Depth Interviews
IR: Implementation Research
NGO: Non-Government Organisation
PHC: Primary Health Centre
VHSNC: Village Health Sanitation and Nutrition Committee
